# Minimally invasive ultrasound-guided thread carpal tunnel release: a video demonstration protocol

**DOI:** 10.1007/s40477-025-01003-0

**Published:** 2025-03-06

**Authors:** Nuno Ferreira-Silva, Tomás Ribeiro-Da-Silva, Lia Lucas-Neto, Keith Aziz, Wesley Troyer, Raúl A. Rosario-Concepción

**Affiliations:** 1 Division of Pain Medicine, Department of Anesthesiology, Reanimation, and Pain Medicine, Hospital Clinic Barcelona, University of Barcelona Calle de Villarroel, 170, 08036 Barcelona, Cataluña Spain; 2 Department of Physical Medicine and Rehabilitation, ULS Santa Maria, Lisboa, Portugal; 3 Department of Anatomy, Faculdade de Medicina, Universidade de Lisboa, Lisboa, Portugal; 4 Department of Orthopedics, Mayo Clinic, Jacksonville, FL USA; 5 Department of Physical Medicine and Rehabilitation, Mayo Clinic, Jacksonville, FL USA; 6 Physical Medicine and Rehabilitation, Baptist Health South Florida, Miami, FL USA

**Keywords:** Carpal tunnel syndrome, Ultrasound-guided procedures, Minimally invasive surgery, Neuropathy

## Abstract

Carpal tunnel syndrome is the most common compressive peripheral neuropathy, often requiring surgical treatment. Recently, ultrasound-guided thread carpal tunnel release has become an attractive minimally invasive alternative to the traditional surgical approaches. This incision-less technique utilizes an abrasive thread and a Tuohy needle to achieve division of the transverse carpal ligament while sparing adjacent soft tissue to decrease post-procedural pain and enhance recovery. Although several step-by-step descriptions of the technique have been published in recent years, no video demonstration of the procedure and the pre-procedure scanning needed for surgical planning exists. This paper aims to provide a video-based guide to ultrasound-guided thread carpal tunnel release and propose a pre-procedure scanning protocol for physicians considering implementing this procedure into their practice.

**Supplementary Information:**

The online version contains supplementary material available at 10.1007/s40477-025-01003-0.

## Introduction

Carpal tunnel syndrome (CTS) is caused by the compression of the median nerve (MN) within the carpal tunnel and is the most common nerve entrapment disorder. It leads to neuropathic pain and affects up to 5% of the population [1]. This condition carries substantial work loss-related costs, with the median time of loss of work at 28 days [2, 3].

Non-surgical management is recommended as the first-line treatment for mild to moderate carpal tunnel syndrome, which includes nocturnal splinting, steroid injections, and nerve hydrodissection [3–5]. Severe cases and those refractory to nonsurgical treatment are typically treated by surgical release. Described techniques for surgical release involve the standard open carpal tunnel release (OCTR), mini-open carpal tunnel release, and endoscopic carpal tunnel release (ECTR) [3, 6, 7]. While ECTR has been promoted as a less invasive procedure, providing reduced postoperative pain and faster return to work compared to OCTR, it carries an increased risk of nerve injury and OCTR remains the standard of care [8–10].

Recently, ultrasound-guided (USG) techniques for dividing the transverse carpal ligament (TCL) have gained popularity. These approaches include the use of hook knives, microblade technology, retractable blades, needle fenestration methods, and the incisionless modified thread carpal tunnel release (TCTR) technique described by Gou et al. (2015 & 2017) [11–16]. This technique uses an abrasive thread looped percutaneously around the TCL that transects the ligament using a back-and-forth, sawing motion. It has been validated by multiple clinical and cadaveric studies demonstrating similar effectiveness, safety, and superiority regarding faster return to work, activities of daily living, and rates of pillar pain compared to OCTR and ECTR while saving costs [16–19].

The growing use of US has led to an increase in USG percutaneous orthopedic procedures, including carpal tunnel release (CTR). This shift has reinforced CTR to move from the operating room to the procedure room or even outpatient settings, prompting numerous step-by-step descriptions of the technique in the literature [20, 21]. However, to our knowledge, no didactic video has been published depicting the steps of the USG TCTR. US has also been found to accurately identify the carpal tunnel (CT) anatomy with its anatomical variability in prior cadaveric studies, making it useful to ensure the candidacy and safety of the procedure [22]. This paper aims to provide a video-based guide to USG TCTR and propose a pre-procedure scanning protocol for physicians considering implementing this minimally invasive procedure into their practice.

## General anatomy

The CT is a fibro-osseous canal situated in the volar wrist. It is bounded posteriorly by the concave volar aspect of the carpal bones, anteriorly by the transverse carpal ligament (TCL), radially by the scaphoid and trapezium, and ulnarly by the pisiform and the hamate. It contains the MN lying superficially to nine flexor tendons—four *Flexors Digitorum Superficialis,* four *Flexors Digitorum Profundus,* and the *Flexor Pollicis Longus* (FPL)*.* The CT narrows distally from its inlet, at the level of the pisiform and scaphoid, towards the outlet, made up of the hamate and trapezium. This configuration might contribute to an increased MN pressure at this level, and explain why incomplete transection of the distal part carries the risk of non-successful TCL release [23, 24].

**Fig. 1:**
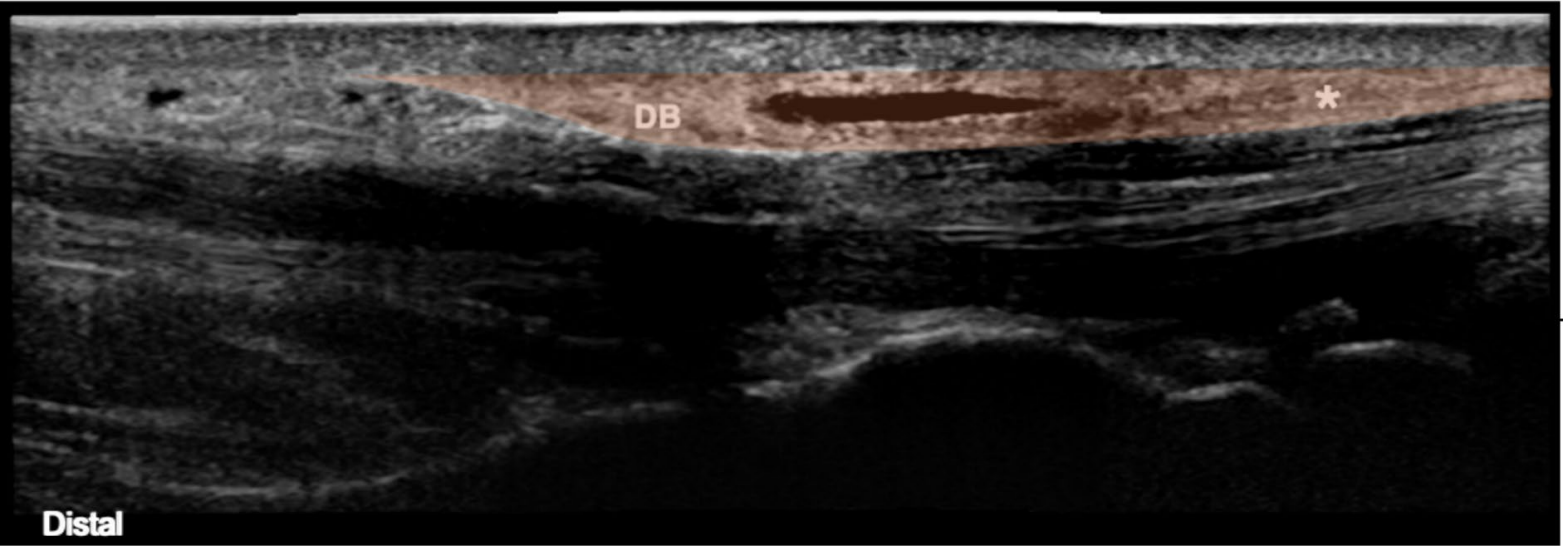
Panoramic view of the transverse carpal tunnel ligament. Legend: *DB* Duck’s beak/Shrimp’s head; *-proximal edge of the transverse carpal tunnel ligament

The TCL is a thin, slightly convex band about 3–4 cm long, thicker in its distal edge (up to 2.5 mm) than the proximal component (up to 0.7 mm), taking the shape of a *duck’s beak or shrimp’s head*, under a US sagittal section [25, 26] (Fig. 1).

Before entering the CT, the MN runs between the *Flexor Digitorum Superficialis* and *Profundus* muscles at the forearm, rises radially to the tendons, and enters the CT superficially slightly radial to the midline [27]. It gives off the palmar cutaneous branch (PCB) about five centimeters proximal to the inlet of the carpal tunnel, which most often (88.3%) arises from its radial aspect [28, 29]. After its origin, the PCB travels between the *Flexor Carpi Radialis* (FCR) tendon and the MN to pierce the antebrachial fascia, typically proximal to the TCL, and continues distally superficial to the ligament. The PCB provides sensory innervation to the skin of the thenar eminence and proximal palm [30].

The second MN branch of interest to TCTR is the recurrent motor branch (RMB). It typically originates from the antero-ulnar aspect of the radial division of the MN at the carpal tunnel outlet and travels vertically in a palmar direction back distal to the TCL to supply the thenar musculature [31].

After exiting the CT, the MN splits into its 3 common palmar digital branches to innervate the sensory component of the first 3 fingers and the radial side of the fourth digit [27].

Finally, the Berrettini branch (Bb), is an anatomic variant described as an anastomosis between the ulnar nerve (UN) and MN, present in up to 60.9% of specimens, most consistently branches from the UN 5 mm distally to the distal border of the TCL and travels radially to join MN, supplying the ulnar side of the third and the radial side of the fourth finger [32, 33].

### Pre-procedure scanning protocol

Pre-procedure systematic evaluation of the US anatomy of the CT is mandatory, as the boundaries and the anatomic variability of the CT structures (described in the next section) must be known and thoroughly defined before intervention. Clinicians should utilize linear high-frequency US probes, typically above 15 MHz, to visualize superficial neurovascular structures and other sonographic landmarks adequately.

The pre-procedure scanning protocol can be divided into three parts:—identification of the MN nerve and its branches;—identification of the inlet and outlet of the carpal tunnel;—and measurement of the transverse and longitudinal safe zones.

We start by positioning the patient’s forearm in full supination and placing the US in the short axis over the MN, approximately 5 cm proximal to the distal wrist crease. Close to this point, in most patients, we can identify the branching of the PCB, seen as a hypoechoic, small nerve fiber coming off the radial side of the MN moving superficially towards the FCR tendon. Following the MN distally, the RMB can be identified as a small C-shaped shadow as it typically will branch off the MN vertically and radially, distal to the TCL in an extraligamentous course before looping back to innervate the thenar musculature [34]. Utmost care should be taken to detect unusual branching patterns/trajectories crossing the working area.

In the second part, we’ll place the probe at the same initial point and scan distally until the CT inlet appears in the field of view (FOV). As mentioned before, the inlet can be identified by its bony landmarks—the scaphoid tubercle and the pisiform, appearing as two rounded hyperechoic structures with posterior acoustic shadowing with the tendon of the FCR and the flattened tendon of *Flexor Carpi Ulnaris* on top, respectively. The MN is seen coursing superficial in the CT parallel to the second and third flexor tendons and ulnar to the FPL tendon. Moving further distally, the two rounded bony landmarks will be replaced, at the outlet, by the sharp-edged hook of the hamate ulnarly, which can be easily identified by the ulnar artery sitting on top, and the rounded surface of the trapezium tubercle deep to the proximal thenar musculature radially. Finally, by gently rotating the probe 90º at the midsection of the TCL, we visualize the proximal and distal TCL edges. Proximally, it has a more linear shape, while distally it presents with the classic *duck’s beak or shrimp head* appearance with the superficial palmar arch (SPA) pulsating distally.

After identifying the outlet and inlet, we measure the working area delimited by the transverse (both at the inlet and outlet) and longitudinal safe zones*.* The transverse safe zone is bounded radially by the median nerve and ulnarly by the ulnar artery or the hook of the hamate, depending on what structure is closer to the nerve [15, 16]. Narrow transverse safe zones can be broadened using the passive ulnar deviation of the wrist. This maneuver increases the distance between the MN and ulnar artery by forcing the flexor tendons against the hook of the hamate. Since the MN is more mobile, it can shift radially with ulnar deviation of the wrist. This increases the distance between the MN and the ulnar artery, which remains fixed in Guyon’s canal [35]. Although no minimal distance has been established as a contraindication for USG TCTR as it largely depends on the proceduralist’s experience and expertise, the procedure becomes exceedingly more challenging in patients with less than 4 mm transverse safe zones, measured with ulnar wrist deviation. For this reason, we typically opt not to perform USG TCTR in these cases. The longitudinal safe zone is defined as the distance between the SPA and distal TCL and serves as the entry point reference. We can adopt a more flexible approach in selecting patients with narrow longitudinal safe zones because saline can be used to hydrodissect the SPA, pushing it distally away from the TCL. This increases the distance between the TCL and the SPA, enhancing procedural safety. Patients with distances as low as 1 mm have been submitted to TCTR with no complications, but ultimately it comes down to the physician’s discretion in each case.

## Anatomic variability

The CT is known to have a variety of neurovascular anatomical variations [36]. Failure to recognize these variations can result in iatrogenic injuries. Since USG TCTR focuses on the CT’s transverse and longitudinal safe zone, only the pertinent neurovascular anatomical variations that could potentially compromise these safety zones will be discussed.

Anatomical narrowing of the transverse safe zone may occur due to variations in the shape/size of the MN and patterns of branching of its nerves. The most frequent variation of the MN is the bifid variant, present in 18.5% of individuals with symptomatic CT [37]. Furthermore, a trifid MN has also been documented [38]. These variants increase the risk of nerve injury by reducing the transverse safe zone but typically do not exclude patients from candidacy. According to Lanz et al. [39], approximately 63% of bifid variants are accompanied by a persistent median artery (PMA), though it can also be found in normal MN in a much lower percentage of cases. This persistent embryonic structure usually runs in between the two fascicles in the case of a bifid MN and on the ulnar side of a single MN [40]. In the latter case, the PMA will lie within the transverse safe zone, making it vulnerable to injury. Similarly, a PCB and RMB originating from the ulnar side of the MN will also result in transverse safe zone narrowing. These variations have been documented in 2% and 5% of cases, respectively [41, 42]. Of note, several authors reported a high variability of the RMB course in individuals with hypertrophic thenar muscles [43, 44]. As such, hypertrophic thenar muscles should prompt a meticulous scan of the RMB course. Furthermore, nerve branches can also be found crossing the transverse safe zone, turning this zone into a “*not so safe*” area. In the case of PCB absence, documented in 10% of cases, the palmar branch of the UN has been found, in some specimens, to cross the transverse safe zone to supply the thenar and proximal palm skin [45, 46]. Additionally, the Berrettini branch can be found inside the CT or at its distal border in 28% of cases [33]. The presence of Berrettini branch variants does not automatically exclude a patient from being a candidate for the procedure. However, it is important to identify these variants so that the Tuohy needle can be inserted before it crosses into the safe zone, preventing them from being looped and transected by the cutting thread.

The longitudinal safe zone can be affected by the variability of the SPA. An incomplete SPA (non-union of the ulnar and radial arteries) or the presence of a PMA may make it challenging to identify the boundaries of the longitudinal safe zone, complicating needle/thread placement. Furthermore, in some individuals, the SPA may lie unusually close to the distal end of the TCL, reducing the available longitudinal safe zone and increasing the risk of vascular damage.

## Contraindications and special considerations

TCTR is considered a safe and effective technique, with studies demonstrating faster clinical improvement, reduced complications, and a shorter return to work compared to open OCTR and ECTR [16, 47]. However, certain contraindications and special cases should be noted.

TCTR is not recommended if pre-procedural scanning can not accurately identify the relevant anatomy mentioned in our scanning protocol, and confirm that no neurovascular structures cross the transverse safe zone. Additionally, as previously stated, a transverse safe zone bigger than 4 mm, with the wrist ulnarly deviated, is advised to ensure the safe positioning of the thread. Moreover, failure to identify the full trajectory of the thread during the procedure imposes an increased risk of complications. In such cases, a conversion to an open release is warranted.

In cases where CTS presents with motor-dominant symptoms due to isolated compression of the recurrent motor branch (RMB), intra-tunnel space-occupying lesions (e.g. ganglion cysts), median artery thrombosis, in cases of amyloidosis requiring biopsies, or in the revision of a previous carpal tunnel release, open carpal tunnel release (OCTR) may be preferred. Similarly, OCTR is recommended in cases where there is a history of sensitivity to the material used in the thread.

## Technical procedure

**Fig. 2:**
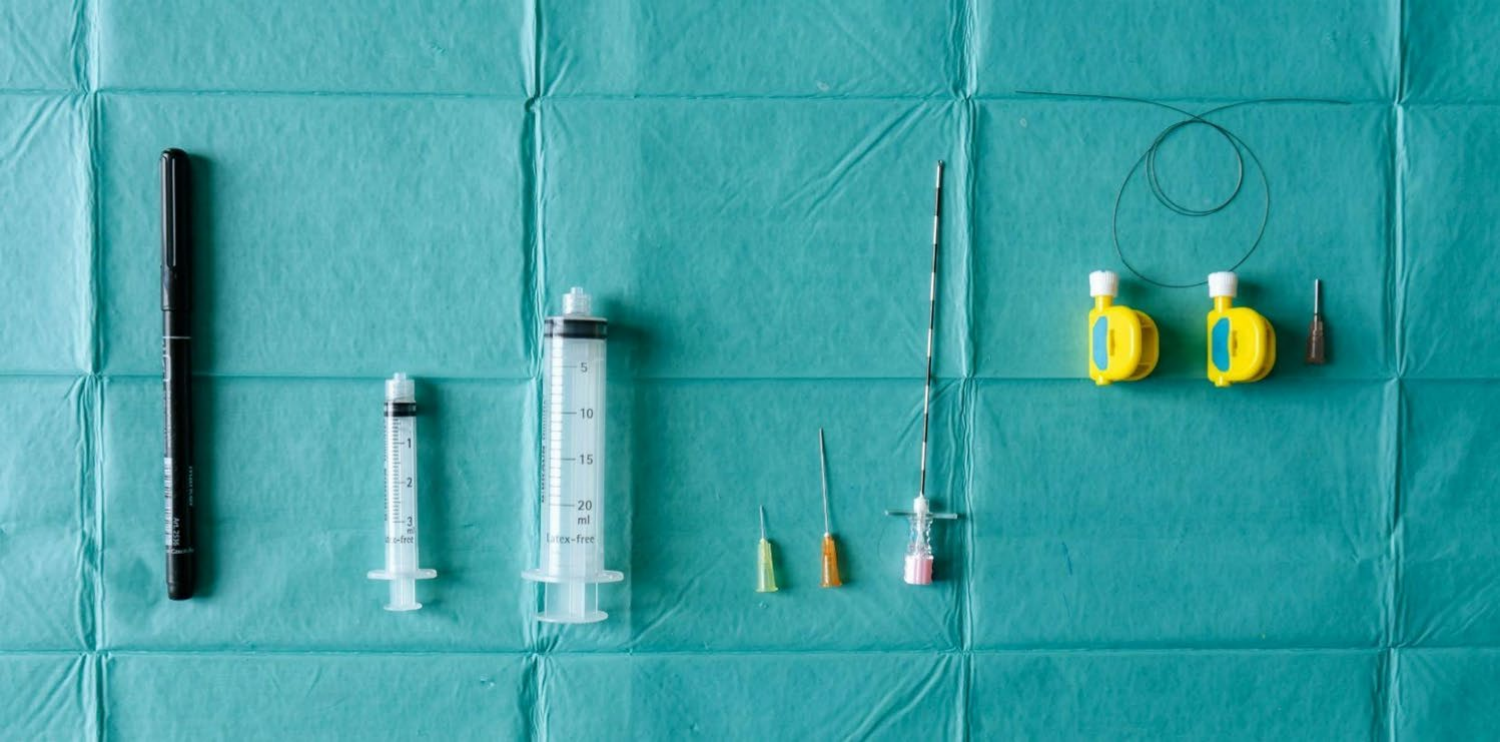
Surgical Instruments. From left to right: Marker; luer lock syringes (3 mL and 20 mL); 30G, 1-inch needle; 25G, 1.5-inch needle; 18G, 4-inch Tuohy needle; surgical dissecting thread (Loop&Shear™, 0.009 inch in diameter; Ridge & Crest Company, Monterey Park, California)*; Epidural Catheter Connector; 18G, 0.5-inch blunt needle. *Courtesy of Danqing Guo and Danzhu Guo

The patient’s arm is placed in the same fully supinated position, resting on an arm board. The instruments needed should be verified using a surgical checklist and the patient’s anatomy of pre-procedure scanning reviewed (Fig. 2).

### Skin marking

After appropriate draping and using a sterile technique, the US probe is placed in the short axis of the CT and a skin marker pen is utilized to mark the midpoint of the transverse safe zone both at the inlet and outlet. A line is then drawn connecting these two points using a ruler and then extended both proximally and distally. This line will serve as a guide for probe and thread orientation during the procedure. Next, the probe is turned 90º degrees to align with the drawn line and moved distally until the SPA and the *duck’s beak/ shrimp’s head* of the distal TLC appear in the FOV (Fig. 1). At this point, the proximal end of the SPA is marked, serving as the distal limit of the entry point. By translating the probe proximally, we mark the exit point, approximately 1 cm proximal to the TCL proximal edge.

### Anesthetic skin wheal (not present in the video)

For local anesthesia, a skin wheal is done with 1% lidocaine at the entry and exit points, using a 30G, 1-inch needle. Approximately 1–2 mL of the anesthetic solution is administered at the entry point, and around 2–4 mL are injected at the needle exit site. Since the exact location of the exit point may not always be precisely predicted, this volume ensures anesthesia of a wider exiting area, as well as anesthetizing the PCB most of the time.

### Hydrodissection

A 25G, 1.5 or 2-inch needle with a 20º–30º bend on a 10 mL luer lock syringe with 1% lidocaine is introduced subcutaneously over the SPA. In cases where the longitudinal safe zone is too narrow, injection of the solution just proximal to the SPA is used to displace the artery distally, augmenting the safe zone. The needle is further advanced in line with the longitudinal line drawn, first between the SPA and the proximal TCL and subsequently between the TCL and the flexor tendons. Injection of the solution will create space between both structures, reducing iatrogenic injury and decreasing discomfort during the procedure.

### Tuohy deep pass

At the same entry point, an 18-gauge, 4-inch Tuohy needle bent 30º at both the 1-cm and 4-cm mark on a 10–20 mL luer lock syringe with saline is introduced. Further hydrodissection is done where needed, and the needle is advanced in the TCL-flexor tendons plane while performing transverse US checks to confirm secure placement. Close to the exit point, surgical forceps or spatula can be used to assist skin perforation. Finally, the cutting thread is passed through the Tuohy needle, which is then removed, leaving the thread in place under the TCL.

### Tuohy superficial pass

Another 18-gauge 4-inch Tuohy needle, bent 15 degrees at the 1-cm mark on a 10–20 mL luer lock syringe with saline is introduced at the point of insertion of the deep thread and advanced while injecting the solution to hydrodissect the TCL-subcutaneous tissue plane. The needle should emerge at the same exit point as the thread. The proximal end of the initially placed thread is inserted back through the new Tuohy needle to create a cutting loop around the TCL. Before the retrieval of the superficial Tuohy needle, the looping of the thread is held outside the skin to ensure placement.

### Thread position verification

A final verification of the thread placement is done to ensure no vascular and nerve structures are at risk of being damaged. The wire is gently wiggled, while US images are checked for movement or tethering of important structures.

### TCL release

**Fig. 3:**
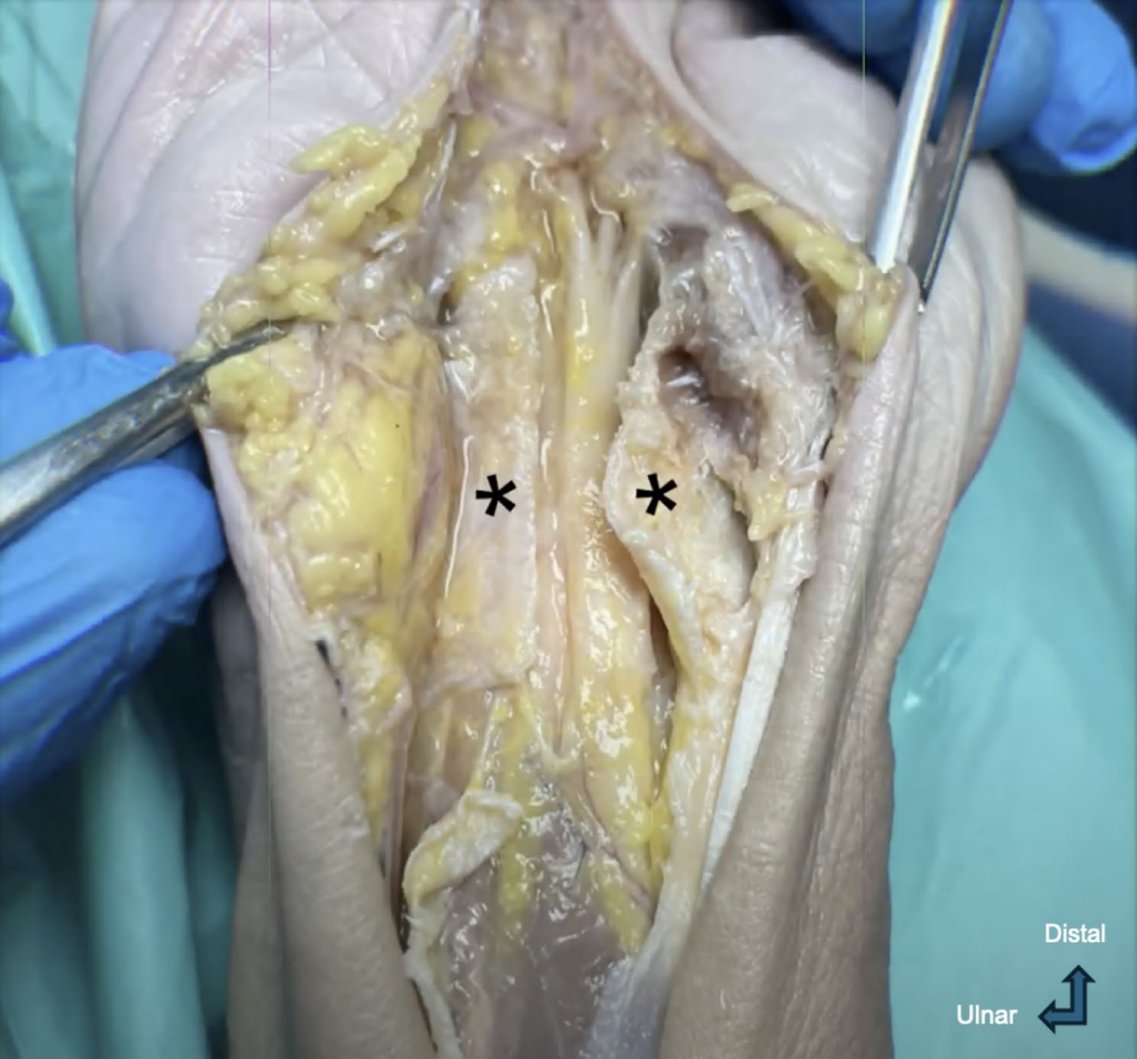
Anatomic dissection (right hand) showing the transverse carpal tunnel ligament divided. Legend: *Ulnar (left) and radial (right) edges of the transected carpal tunnel ligament

After the safety checks, a protective 18G, 0.5-inch blunt needle is positioned at the entry site, ensuring that both ends of the thread pass through it. This protective measure will prevent laceration of the palmar skin, by securing the approximation of the cutting threads and acting as a protective barrier. With the patient’s hand secured, the thread ends are pulled distally, employing a back-and-forth sawing motion, dividing the TCL. This motion should be continued until the TCL is completely transected and the thread exits through the entry point intact, ensuring no structural failure of the thread has occurred (Fig. 3).

## Videos

Please refer to Online Supplemental Video 1 (accompanying this manuscript) for the US pre-procedure protocol scan in a live model. Please refer to Online Supplemental Video 2 (accompanying this manuscript) for a step-by-step demonstration of the USG TCTR in a cadaveric specimen.

## Conclusion

USG TCTR is a novel, minimally invasive technique requiring physicians to possess advanced knowledge of the CT anatomy and its neurovascular variants, along with high-level expertise in US-guided procedures. High-frequency US machines should also be utilized to improve the visualization of small neurovascular structures. Overall, these prerequisites render the TCTR a safe and feasible procedure for physicians that could shift the paradigm from open CT releases to office-based USG TCTR.

## Supplementary Information

Below is the link to the electronic supplementary material.

Supplementary file1 (MP4 49664 KB)

Supplementary file2 (MP4 97942 KB)

Supplementary file3 (PNG 229 KB)

Supplementary file4 (PNG 301 KB)

Supplementary file5 (DOCX 14 KB)

Supplementary file6 (DOCX 16 KB)

## Data Availability

Not applicable.
