# Supplementary figures and images for: Minimally invasive ultrasound-guided thread carpal tunnel release: a video demonstration protocol
Source: J Ultrasound. 2025 Mar 6;28(4):803–10. doi: 10.1007/s40477-025-01003-0 (PMC12675869; doi:10.1007/s40477-025-01003-0)

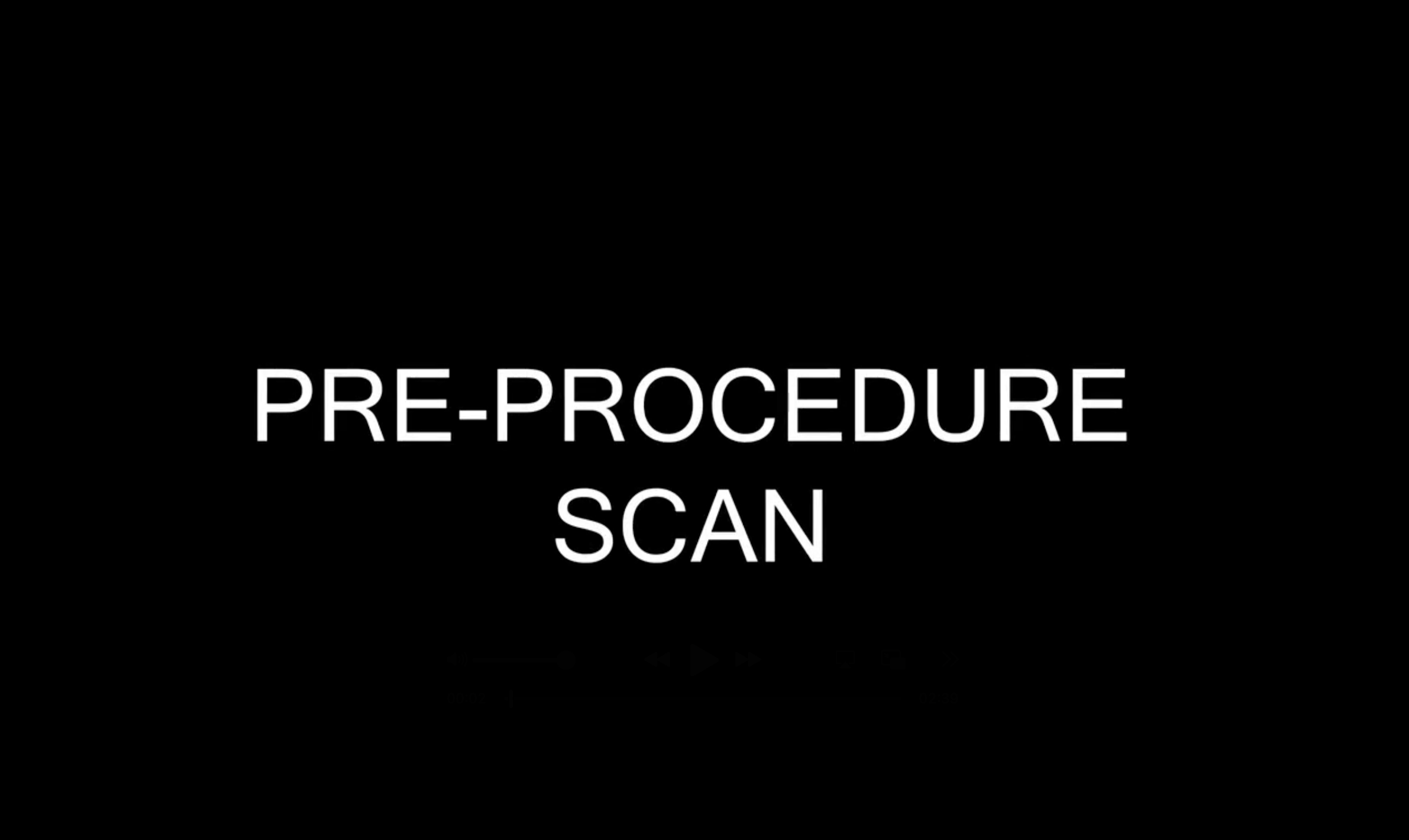

Supplement: Supplementary file 3 — Supplementary file3 (PNG 229 KB) [file 40477_2025_1003_MOESM3_ESM.png]

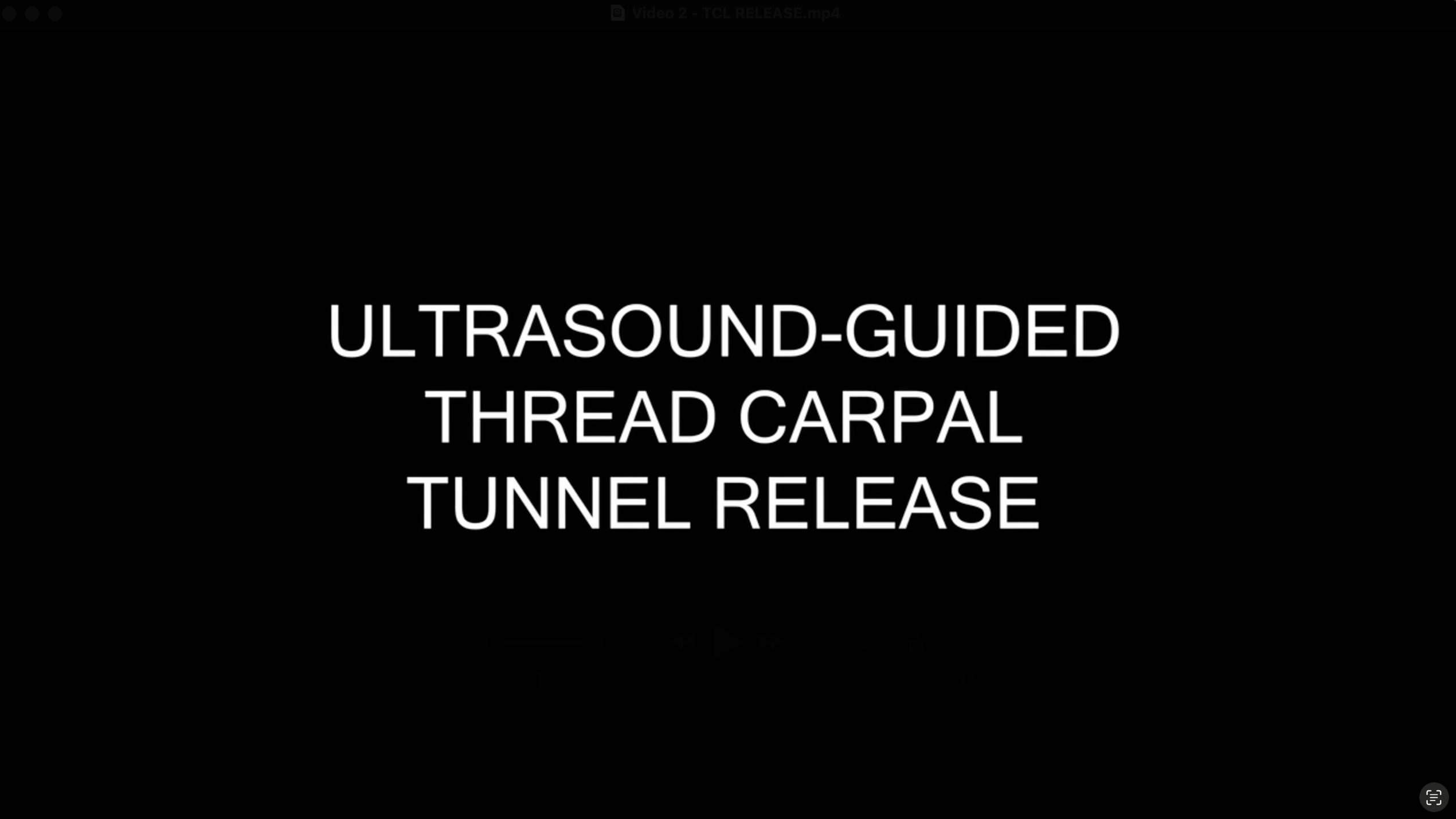

Supplement: Supplementary file 4 — Supplementary file4 (PNG 301 KB) [file 40477_2025_1003_MOESM4_ESM.png]
