# Supplementary material for: Minimally invasive ultrasound-guided thread carpal tunnel release: a video demonstration protocol
Source: J Ultrasound. 2025 Mar 6;28(4):803–10. doi: 10.1007/s40477-025-01003-0 (PMC12675869; doi:10.1007/s40477-025-01003-0)
Supplement: Supplementary file 5 — Supplementary file5 (DOCX 14 KB) [file 40477_2025_1003_MOESM5_ESM.docx]

**Video 1 Manuscript: PRE-PROCEDURE SCAN**

We start the pre-procedure scan by identifying the median nerve and its main branches.  The median nerve is identified approximately 5 cm proximal to the wrist with its palmar cutaneous branch, typically arising from the radial aspect of the nerve, shown here.

By moving the probe back and forth we can clearly see the branching of the palmar cutaneous nerve and its superficial course.

Going further distally we will see the thenar eminence, and just below it we will identify the motor thenar branch, moving vertically in a palmar direction - shown here. It is essential to ensure that this branch travels on the radial side of the median nerve, as anatomic variations may occur.

To identify the inlet of the carpal tunnel, we trace the median nerve from the distal forearm caudally until two bony landmarks become visible: the scaphoid on the radial side and the pisiform on the ulnar side, as seen here.

As we continue distally, the outlet of the carpal tunnel is identified by two other bony landmarks the trapezium on the radial side and the hook of the hamate on the ulnar side.

We proceed to measure the transverse safe zone at the inlet. We measure the distance between the ulnar end of the median nerve and the radial end of the ulnar artery. This distance increases with passive ulnar wrist deviation. In this particular case, it doubles.

The same measurement is performed at the outlet, assessing the distance between the median nerve and the ulnar artery. Similarly, the transverse safe zone also expands with passive ulnar wrist deviation, although less pronounced here.

By turning the probe 90º we will now measure the longitudinal safe zone.

This zone is delimited by the superficial palmar arch complex distally and the caudal end of the transverse carpal ligament proximally.
