# Supplementary material for: Minimally invasive ultrasound-guided thread carpal tunnel release: a video demonstration protocol
Source: J Ultrasound. 2025 Mar 6;28(4):803–10. doi: 10.1007/s40477-025-01003-0 (PMC12675869; doi:10.1007/s40477-025-01003-0)
Supplement: Supplementary file 6 — Supplementary file6 (DOCX 16 KB) [file 40477_2025_1003_MOESM6_ESM.docx]

**Video 2 Manuscript: PROCEDURE**

We start by identifying the median nerve proximal to the transverse carpal ligament and trace the nerve distally until the proximal carpal tunnel. At this point, we identify the transverse safe zone between the median nerve and the ulnar artery.

After identifying the median nerve and ulnar artery, a mark is drawn in the middle of the transverse safe zone, first at the inlet of the carpal tunnel, and then distally at the carpal tunnel outlet.

Once both midpoints are identified, we draw a line connecting the two points and extend it proximally and distally to the transverse carpal ligament. This line will serve as a crucial reference for the placement of the threads.

The ultrasound probe is rotated 90 degrees to obtain a longitudinal view of the flexor tendons. In this position, we identify the distal transverse carpal ligament and mark the superficial palmar arch. Accurately marking the SPA safeguards against vascular injury and serves as an entry point reference.

The exit point is also drawn about 1 centimeter proximal to the end of the transverse carpal ligament.

The distal line marks the SPA, while the proximal line marks the exit point.

Both entry and exit points are anesthetized.

After identification of the superficial palmar arch and the distal end of the transverse carpal ligament, a 25-gauge, 1-inch needle is introduced between these two structures and advanced deep to the transverse carpal ligament. Hydrodissection of the plane between the transverse carpal ligament and the flexor tendons is performed by injecting 1% lidocaine creating a safe space for needle passage. Typically, 5 to 10 milliliters of 1% lidocaine are used.

After hydrodissection, a 18-gauge, 4-inch Tuohy needle bent 30 degrees at both the 1-centimeter and 4-centimeter mark is introduced. The needle is advanced proximally within the carpal tunnel while injecting saline to further hydrodissect the transversal carpal ligament - flexor tendons plane. The midline of the transverse safe zone drawn on the skin is used to position the ultrasound probe, ensuring that the Tuohy needle aligns with it between the median nerve and the ulnar artery.

Additionally, a 90 degrees rotation of the ultrasound probe to the tranverse view of the tendons is used to confirm secure needle advancement.

The Touhy needle finally pierces the skin at the exit point.

The cutting thread is passed through the Tuohy needle and the needle is withdrawn, leaving the thread positioned deep to the transverse carpal ligament, within the transverse safe zone of the carpal tunnel.

After identification of the deep thread, a second 18-gauge 4-inch Tuohy needle, bent 15-degree angle at the 1-centimeter mark, is inserted at the entry point and advanced, this time superficially to the transverse carpal ligament until its tip emerges at the same exit point.

The proximal end of the initially placed thread is inserted back through the new Tuohy needle to create a cutting loop for the Transverse Carpal Ligament. Before the retrieval of superficial Touhy needle the looping of the thread is held outside the skin.

After placing of both threads. the wrist is scanned to ensure the threads slide freely away from the median and ulnar artery.

Before the initiation of the release, both threads are inserted in a 0,5-inch 18-gauge blunt needle at the entry site to protect the skin. The two thread ends are then pulled distally using a back-and-forth sawing motion until the transverse carpal ligament is fully transected, allowing the thread to exit through the entry point.
